# A case report of integrating Chinese and Western medicine: A new era in the treatment of late-onset Krabbe disease

**DOI:** 10.1097/MD.0000000000046947

**Published:** 2026-01-09

**Authors:** Mengyao Tang, Hui Liu, Luqiao Che, Liping Zhang

**Affiliations:** a Zhejiang Chinese Medicine University, The First School of Clinical Medicine, Hangzhou, Zhejiang Province, China; b The First Affiliated Hospital of Zhejiang Chinese Medical University (Zhejiang Provincial Hospital of Traditional Chinese Medicine), Hangzhou, Zhejiang Province, China.

**Keywords:** case report, Chinese medicine, Krabbe disease

## Abstract

**Rationale:**

Krabbe disease is a rare autosomal recessive genetic disorder for which no specific cure is currently available. In clinical practice, management primarily relies on supportive and symptomatic therapies aimed at alleviating patient suffering. Although the disease remains incurable, the intensity of medical interventions has decreased over time, highlighting the need for innovative and integrated therapeutic approaches. This case study presents a patient who received a combination of Western medicine and traditional Chinese medicine, resulting in a significant reduction in clinical symptoms.

**Patient concerns:**

The patient primarily presents with unsteady gait, bilateral lower extremity weakness, and nocturnal muscle tremors in both lower limbs. Electromyography examination revealed functional impairment of the deep sensory pathways in both lower extremities. Additionally, the mutation sites identified in this patient are c.1901T>C and c.98T>G; notably, the mutation site c.98T>G has not been previously reported. The therapeutic efficacy of Western medicine for this condition appears to be unsatisfactory.

**Diagnoses:**

This patient was diagnosed with Krabbe disease.

**Interventions:**

On January 17, 2025, the patient decided to incorporate traditional Chinese medicine into the treatment regimen for concurrent therapy.

**Outcomes:**

During the concurrent treatment with integrated traditional Chinese and Western medicine, the patient exhibited remission of clinical symptoms and a corresponding improvement in quality of life.

**Lessons:**

The integrated treatment of traditional Chinese and Western medicine offers new therapeutic options for the management of Krabbe disease.

## 1. Introduction

### 1.1. Overview

Krabbe disease (KD), also known as globoid cell leukodystrophy, is a rare autosomal recessive genetic disorder characterized by lysosomal accumulation. Specifically, mutations in the gene encoding lysosomal galactosylceramidase (GALC) lead to this lysosomal accumulation.^[1,2]^ Additionally, GALC deficiency can result in the accumulation of its substrate, galactosylsphingosine (commonly referred to as psychosine), in the brain.

This further leads to rapid and severe neurodegeneration, neuroinflammation, and demyelinating changes in the central and peripheral nervous systems.^[3]^ The GALC gene is located on chromosome 14q31. To date, the Human Gene Mutation Database has confirmed that there are 275 distinct mutations in genes associated with Krabbe disease.^[4]^

Clinically, this disease is typically classified into early-onset (infantile) and late-onset types based on the age of onset. The late-onset type encompasses late infancy, late childhood, adolescence, and adulthood.^[5]^ Clinical symptoms and prognosis are closely associated with the age of onset. For the early-onset (infantile) type, affected infants usually develop the disease rapidly within 3 to 6 months of age, presenting with irritability, intermittent crying, feeding difficulties, and rapidly progressing to dystonia, muscle spasms, visual impairment, and other symptoms. Due to the rapid disease progression, most of these infants die within 1 to 3 years.^[6]^ Infants with late-onset disease (onset in late infancy) and patients with adolescent-onset disease often present with symptoms such as ataxia, vision loss, and psychomotor regression.^[7]^ In terms of survival time among infant patients, those with late-onset (late infancy) disease have a significantly longer lifespan (2 to 14 years) compared to those with early-onset (infantile) disease.^[8]^However, adult-onset disease, a subset of the late-onset type, is relatively rare in clinical practice. It is typically characterized by slowly progressive spastic paraparesis, bradykinesia, gait abnormalities, and may also be accompanied by neurological impairment symptoms such as cognitive dysfunction and bulbar palsy.^[8,9]^

Following screening conducted as part of the 2006 New York State Census in the United States, the estimated incidence of Krabbe disease was found to be 1 case per 100,000 individuals, with the early infantile phenotype accounting for over 90% of cases. Currently, there are no available epidemiological data on KD in China.^[10]^

Due to the clinical heterogeneity of KD, adult patients exhibit significant variability in both clinical symptoms and disease progression. Atypical cases are frequently misdiagnosed and thus left untreated. Currently, diagnosis primarily relies on the detection of GALC activity in the blood. Specifically, a GALC activity level lower than 5% of the normal mean (4.2 nmol/h/mg protein) is considered a positive result. However, this diagnostic method has relatively low specificity: it is susceptible to interference from multiple factors, and its results are not entirely reliable.

Therefore, in clinical practice, in addition to clinical symptoms, neuroelectrophysiological assessments, and imaging studies, molecular genetic testing serves as a reliable criterion for the diagnosis of this disease.^[11]^

At present, there is no specific treatment for this disease. To alleviate patient suffering, symptomatic and supportive treatments are predominantly utilized in clinical practice. Hematopoietic stem cell transplantation, enzyme replacement therapy, virus-mediated gene therapy, and substrate reduction therapy (e.g., L-cycloserine) represent promising emerging therapeutic directions for the future.^[12]^Unfortunately, symptomatic treatment cannot fully relieve patients’ suffering. In adult patients, bradykinesia, limb stiffness, and abnormal sensations can significantly impair quality of life and contribute to a spectrum of emotional difficulties.

### 1.2. Case review

**Figure 1.**
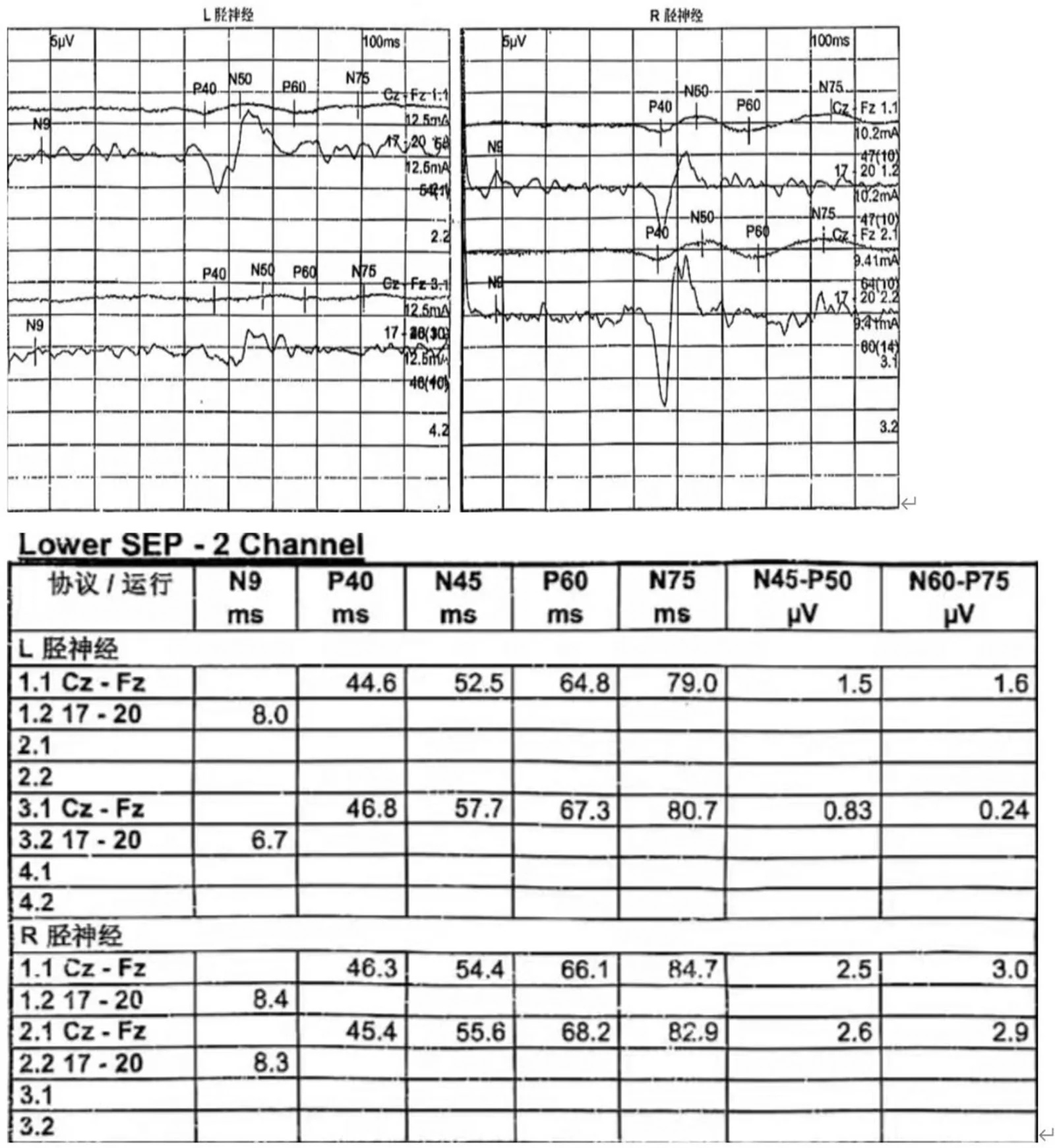
Electromyography report of the patient.

**Figure 2.**
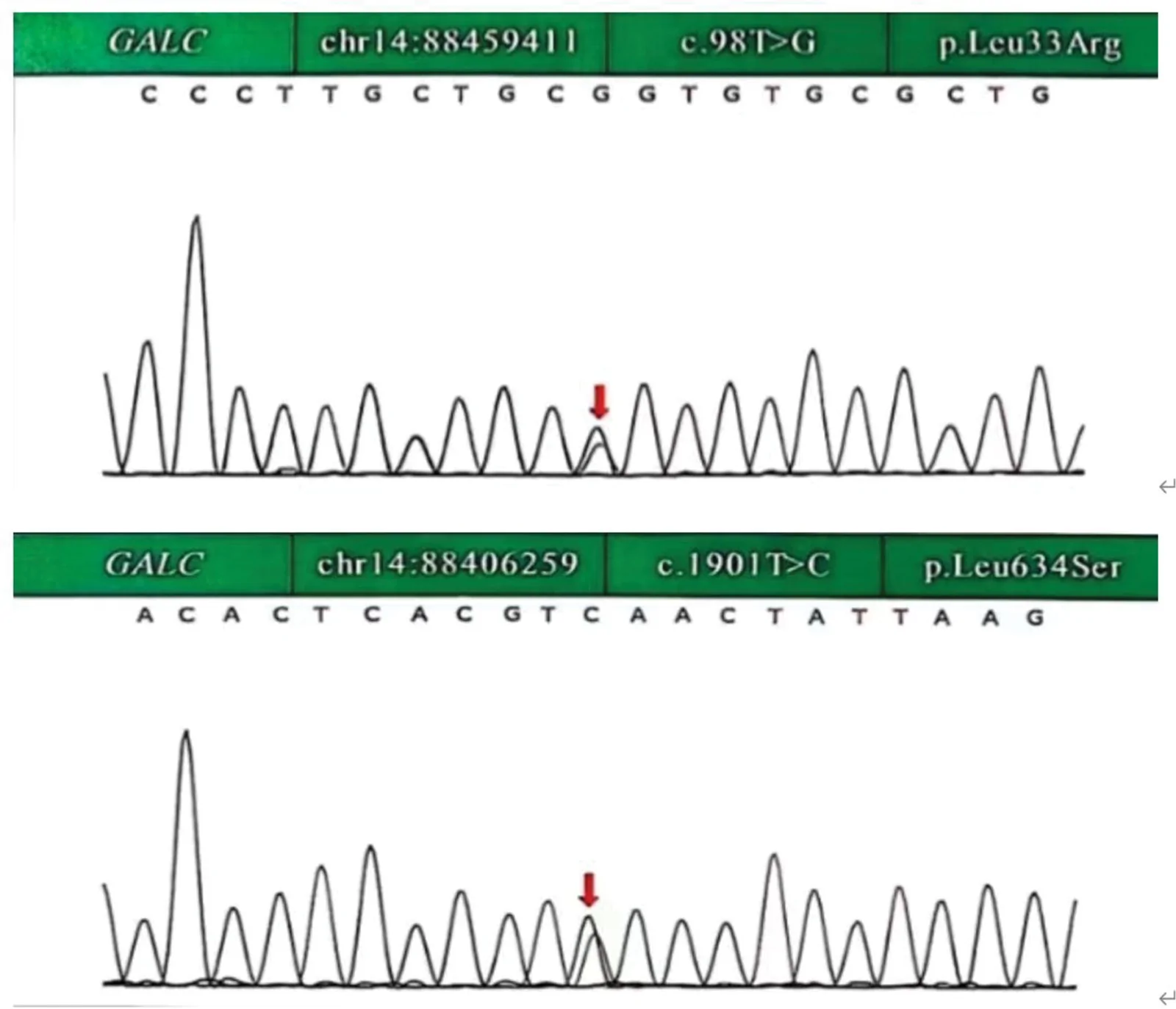
The patient’s genetic test report.

This 54-year-old male patient had a past medical history of pulmonary nodules, right-sided thyroid nodules, fatty liver, and hepatic cysts, as well as a history of smoking and alcohol consumption. The patient had no significant preference for alcohol consumption but occasionally consumed alcohol unavoidably in work-related social settings. Regarding the family history, the patient’s elder sister and elder brother both had a history of KD, with manifestations of limb spasm and unsteady gait during the onset. In 2023, the patient first developed unsteady gait, with an increased tendency to fall when walking on uneven surfaces or after alcohol intake. One year prior to presentation, the patient reported a progressive worsening of gait instability compared to baseline. He experienced difficulty standing up after prolonged sitting, bilateral lower limb weakness, right foot elevation difficulty, and bilateral foot numbness, soreness, and distension. Nocturnal continuous muscle fasciculations were noted, occurring approximately 3 times per minute; in severe cases, these symptoms impaired the patient’s sleep quality.The patient was admitted to the First Affiliated Hospital of Zhejiang University School of Medicine (also known as Zhejiang First Hospital) in July 2024. On physical examination, bilateral lower limb sensory hypesthesia and a positive Babinski Sign were noted. Additionally, the patient exhibited gait instability and difficulty walking in a straight line; the remainder of the physical examination was unremarkable. No significant abnormalities were detected in thyroid function tests, tumor marker panels, anti-neutrophil cytoplasmic antibodies, or antinuclear antibody panels. Ambulatory electroencephalography revealed no abnormalities in the power of each frequency band across all brain regions. Electromyography for suspected peripheral neuropathy of the upper and lower limbs demonstrated functional impairment of the deep sensory pathways in both lower extremities (involving the popliteal fossae and the proximal/distal segments of the lower limbs, on average). The patient’s Electromyography findings are presented in Figure 1.Cranial magnetic resonance imaging AD sequence of the patient revealed hyperintense T2-weighted signals in the bilateral frontal and parietal lobe subcortical regions, periventricular areas, and centrum semiovale white matter. Based on the aforementioned clinical symptoms, signs, and diagnostic findings, a definitive diagnosis could not be established. Given the patient’s positive family history, genetic testing was recommended. The patient’s genetic test results are presented in Figure 2. These results demonstrated 2 heterozygous variants in the GALC gene (associated with Krabbe disease), namely c.1901T > C and c.98T > G. Subsequent family genetic testing confirmed that the GALC gene mutation sites of the patient’s elder sister are c.1901T > C and c.98T > G, those of the patient’s elder brother is c.98T > G, and those of the patient’s daughter is c.1901T > C.Notably, c.1901T > C is a common mutation site in Asian patients with adult-onset KD,^[13]^ while the other variant, c.98T > G, has not been previously reported.

The patient’s Western medical treatment course was as follows: intravenous mecobalamin for neurotrophic support (administered July 27 to August 1, 2024), plus oral tizanidine (2 mg 3 times daily), clonazepam (0.5 mg once nightly), and gabapentin (300 mg once nightly). The patient was discharged on August 2, 2024. Post-discharge, the patient continued the aforementioned oral medications at the same doses for 2 weeks before discontinuing them. Following this treatment regimen, the patient reported dissatisfaction with the therapeutic outcome. Persistent symptoms included gait instability, bilateral lower limb weakness with difficulty in limb elevation, and unrelieved numbness, soreness, and distension. Nocturnal continuous muscle fasciculations caused frequent nighttime awakenings; upon awakening, the patient had difficulty falling back asleep, which severely impacted daily life and work. On January 17, 2025, the patient initiated concurrent traditional Chinese medicine (TCM) treatment for the first time. At this stage, Western medical management consisted of mecobalamin (0.5 mg twice daily) combined with vitamin and folic acid supplementation.The frequency of alcohol consumption due to work and social needs remained roughly consistent for the patient before and after integrated traditional Chinese and Western medicine (TCM-WM) treatment.

### 1.3. Chinese medicine intervention process

On January 17, 2025, the patient initiated integrated TCM-WM treatment. At the initial consultation, based on the patient’s reported symptoms, including gait instability, bilateral lower limb weakness, and persistent muscle fasciculations, the TCM diagnosis was formulated in accordance with TCM theory: the kidney essence is closely associated with bone development and nourishment, which may alleviate the patient’s aforementioned limb weakness. Additionally, TCM holds that the liver is intimately linked to the tendons and meridians; when the tendons and meridians maintain a balance of contraction and relaxation, normal muscle function is sustained.Furthermore, liver-stored blood also nourishes the body’s muscles. A deficiency in liver essence leads to inadequate nourishment of the tendons and meridians, resulting in muscle fasciculations. The patient’s clinical manifestations, pale red tongue with a yellow, greasy coating and a deep pulse, were indicative of internal retention of dampness-heat. In summary, the patient’s TCM syndrome pattern was diagnosed as liver-kidney deficiency complicated by dampness-heat. Consequently, the TCM treatment principle was determined to be “nourishing the liver and kidneys while clearing internal dampness-heat.” Guided by this principle, an appropriate herbal formula was selected; the herbs were decocted in water for 30 minutes to extract their active components, yielding a liquid preparation. The patient’s administration regimen was 150 mL per dose, twice daily (at 8:00 AM and 4:00 PM). The detailed herbal composition is provided in Table 1. Beyond the 2 core herb categories (i.e., those tonifying the liver and kidneys, and those clearing internal dampness-heat), the formula also included symptom-targeted herbs to address specific complaints: dispelling wind, dredging collaterals, improving appetite, and enhancing sleep.

**Table 1:** Chinese herbalmedicine prescription.

Classification in traditional Chinese medicine	Compositions in Chinese name	Compositions in Latin name	Daily dosage	Modern pharmacology
Tonifying liver And-kidney Medicine	Shudihuang	Rehmannia glutinosa	15 g	Anti-osteoporosis,^[14]^ regulation of inflammatory factors,^[15]^ neuroprotection^[16]^ etc
	Niuxi	Achyranthesbidentata-Blume	30 g	Immunoenhancement, prevention of neuronal apoptosis^[17]^
	Duhuo	Heracleum-hemsleyanum Diels	12 g	Analgesia and anti-inflammation,^[18]^ alleviation of nerve inflammation^[19]^
	Jisheng	Viscum coloratum	15 g	Anti-inflammation, analgesia^[20]^
Eliminate damp-heat Medicine	Yiyiren	Coix lacryma-jobi	15 g	Reduction of excessive fluid absorption by the intestinal lumen^[21]^
	Cangzhu	Atractylodes Lancea	12 g	Reduction of cellular inflammatioz^[22]^ Antitumor activity^[23]^
	Huangbai	Phellodendron amurense Rupr	12 g	Gastric mucosal protection,^[24]^ Neuroprotection^[25]^
“Shujin-Huoluo” Medicine	Gegen	Puerariae-Lobatae Radix	30 g	Antioxidative and immunoregulatory effects^[26]^
	Mugua	Chaenomelis Fructus	30 g	Analgesic,anti-inflammatory,and immunopotentiating effects^[27]^
	shaoyao	Paeonia lactiflora Pall	15–40 g	Analgesic and immunomodulatory effects^[28]^
“Qufeng Tongluo” Medicine	Quanxie	Scorpion	6 g	Neuropathic pain treatment and antiepilepticactivity,^[29,30]^immunoregulatory effects^[31]^
	Chantui	Cicadae Periostracum	6 g	Anticonvulsant, sedative, and hypothermic effects^[32]^
	Jiangcan	Bombyx batryticatus	12 g	Anticoagulant effects^[33]^ and anticancer activity^[34]^
“HuatanHewei” medicine	Sharen	Chaenomelis Fructus	6 g	Anti-inflammatory effects,^[35]^ antibacterial effects, and microbiota regulatory effects^[36]^
	Banxia	Pinellia ternate	12 g	Antitussive, expectorant, and antiemetic effects^[37]^
	Beishumi	Oryza-sativa-varglutinosa	15 g	Anti-inflammatory effects, sleep improvement, and neuronal cell repair^[38]^
	Gancao	Glycyrrhiza uralensis	9 g	Anti-inflammatory, antiviral, antioxidative, and neuroprotective effects^[39]^
follow-up “BushenQianggu” Medicine	Xixiancao	Siegesbeckia orientalis L	15 g	Anti-inflammatory, antithrombotic, and immunosuppressive effects^[40]^
	Jixueteng	Callerya-reticulata (Benth) Schot	15 g	Antiviral, antioxidative, and analgesic effects^[41]^
	Haifengteng	Piper kadsura	15 g	Anti-inflammatory and analgesic effects^[42]^
	Guijia	Chinemys reevesii	15 g	Anti-osteoporotic, hematopoietic, and cerebral tissue protective effects^[43]^
	Qiannianjian	Homalomena occulta	15 g	Anti-inflammatory, analgesic,^[44]^ and anti-osteoporotic effects^[45]^
	Shenjincao	Lycopodium- japonicum	15 g	Antioxidative effect^[46]^
“ZhongZhenAnShen” Medicine	Zishiying	Fluoritum	30 g	Sleep duration prolongation^[47]^
	Zibeichi	-	30 g	Sleep duration prolongation^[47]^
Eliminate damp-heat Medicine	Huangbai	Cortex-Phellodendri Chinensis	12 g	Improvement of intestinal flora, antidiarrheal effects,^[48]^ and bacteriostatic activity^[49]^
“YangXueQuFeng” Medicine	Danggui	*Angelica- sinensis* (Oliv) Diels	12 g	Antiplatelet aggregatory, hematopoietic, and blood-replenishing effects^[50]^
	Fangfeng	Saposhnikovia-divaricata	6 g	Antipyretic,analgesic,^[51]^and immunopotentiating effects^[52]^
“HuaShiHeWei” Medicine	Maiya	Fructus-Hordei-Germinatus	15 g	Anti-colitic^[53]^and intestinal flora regulatory effects^[54]^

### 1.4. Results

Three weeks after initiating TCM preparation treatment, corresponding to the patient’s third outpatient follow-up, bilateral lower limb muscle tone was reduced compared to baseline, though persistent muscle fasciculations and concurrent sleep disturbances remained. As documented in the patient’s outpatient follow-up records (Table 2), following the alleviation of internal dampness-heat in the later treatment phase, the types and dosages of liver- and kidney-nourishing herbs were increased, and symptom-targeted herbs were added. By the sixth outpatient follow-up, the patient’s nocturnal muscle fasciculations had significantly decreased compared to prior assessments. Concurrently, the patient exhibited improved ability to lift the right lower limb and enhanced sleep quality, enabling the resumption of normal daily activities and work.

**Table 2:**
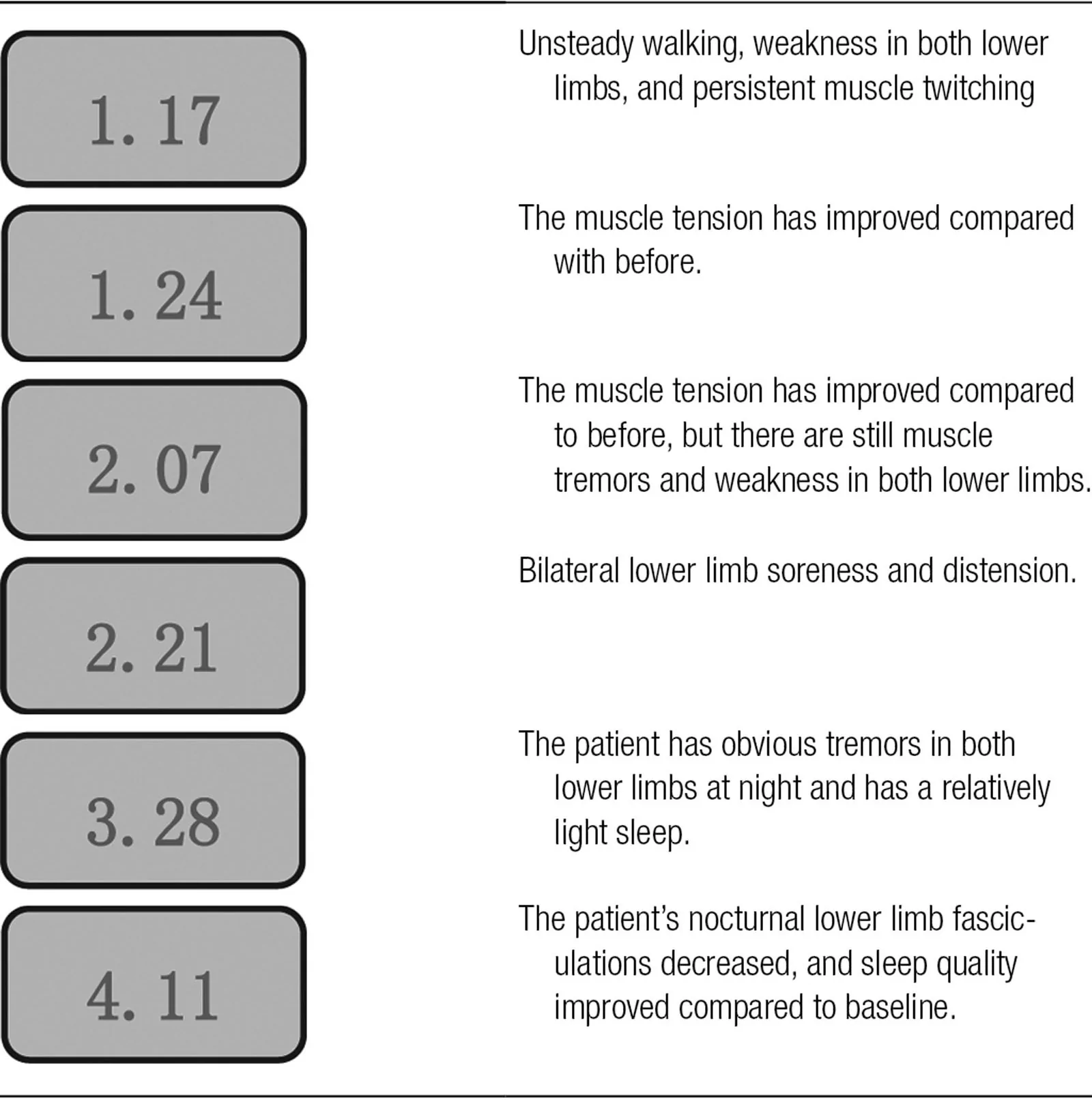
Patient’s outpatient follow-up record.

## 2. Discussion

In TCM theory, there is no direct equivalent term for this disease. Clinically, based on the patient’s presenting symptoms of bilateral lower limb weakness and gait instability, this condition is typically categorized under the TCM concept of “flaccidity disease” (Wei syndrome). Once the TCM condition classification is established, syndrome differentiation is performed based on the patient’s symptoms, tongue signs, and pulse manifestations. Subsequently, therapeutic principles are determined according to the identified syndrome, which in turn guide clinical medication. Per TCM theory, the human body functions as an integrated whole; any internal pathological changes can manifest externally through indicators such as the patient’s subjective symptoms, tongue coating alterations, and pulse variations. This embodies the core TCM principle of “holism” (the holistic concept of the human body).The rationale for syndrome differentiation and the corresponding treatment approach for this patient have been outlined in the preceding sections. Additionally, numerous clinical and pharmacological studies have elucidated the mechanisms through which certain Chinese herbal medicines alleviate patient symptoms and exert therapeutic effects. While the pharmacological actions of multi-herb combinations remain incompletely understood at present, this gap provides a direction for future research. To our knowledge, this represents the first report on the treatment of KD with TCM. As confirmed by the patient, following 3 months of treatment, their distressing symptoms had significantly improved compared to baseline.

Given the current lack of specific treatments for this disease, TCM-guided TCM therapy has demonstrated clear efficacy in alleviating patient symptoms. This approach thus offers a novel therapeutic strategy for managing the condition.

### 2.1. Limitations of the report

As this constitutes a single case report of KD, it inherently has certain limitations. Additionally, in clinical practice, treatment response is typically evaluated based on patient-reported outcomes, which introduces subjectivity into our assessments. Meanwhile, during the treatment process, due to the lack of a control group, we cannot rule out the impact of the placebo effect on the patients.To address these limitations, future research should include Large-Scale Prospective Randomized Controlled Trial and the development of validated, precise assessment scales to more authentically evaluate the efficacy of traditional Chinese medicine and quantify patients’ clinical improvements.

## Author contributions

**Writing – original draft:** Mengyao Tang, Hui Liu.

**Writing – review & editing:** Luqiao Che, Liping Zhang.

## Correction

This article was originally published with an incorrect affiliation “a” which has been now updated online from “a The First School of Clinical Medicine, Zhejiang University of Chinese Medicine, Hangzhou, Zhejiang Province, China” to “a Zhejiang Chinese Medicine University, The First School of Clinical Medicine, Hangzhou, Zhejiang Province, China.”

## References

[1] Kolodny EH Raghavan S Krivit W. Late-onset Krabbe disease (globoid cell leukodystrophy): clinical and biochemical features of 15 cases. Dev Neurosci. 1991;13:232–9. 10.1159/000112166 1817026

[2] Wenger DA Rafi MA Luzi P. Molecular genetics of Krabbe disease (globoid cell leukodystrophy): diagnostic and clinical implications. Hum Mutat. 1997;10:268–79. 10.1002/(SICI)1098-1004(1997)10:4<268::AID-HUMU2>3.0.CO;2-D 9338580

[3] Feltri ML Weinstock NI Favret J Dhimal N Wrabetz L Shin D. Mechanisms of demyelination and neurodegeneration in globoid cell leukodystrophy. Glia. 2021;69:2309–31. 10.1002/glia.24008 PMC8502241 33851745

[4] Zhang C Liu Z Dong H. Two cases of female Chinese adult-onset krabbe disease with one novel mutation and a review of literature. J Mol Neurosci. 2021;71:1185–92. 10.1007/s12031-020-01742-1 33190188

[5] Hagberg B. Krabbe’s disease: clinical presentation of neurological variants. Neuropediatrics. 1984;15(Suppl):11–5. 10.1055/s-2008-1052374 6546163

[6] Suzuki K Suzuki K. Genetic galactosylceramidase deficiency (globoid cell leukodystrophy, Krabbe disease) in different mammalian species. Neurochem Pathol. 1985;3:53–68. 10.1007/BF02834075 3895053

[7] Suzuki K. Globoid cell leukodystrophy (Krabbe’s disease): update. J Child Neurol. 2003;18:595–603. 10.1177/08830738030180090201 14572137

[8] Barone R Brühl K Stoeter P Fiumara A Pavone L Beck M. Clinical and neuroradiological findings in classic infantile and late-onset globoid-cell leukodystrophy (Krabbe disease). Am J Med Genet. 1996;63:209–17. 10.1002/(SICI)1096-8628(19960503)63:1<209::AID-AJMG37>3.0.CO;2-Q 8723112

[9] Wu G Li Z Li J. A neglected neurodegenerative disease: adult-onset globoid cell leukodystrophy. Front Neurosci. 2022;16:998275. 10.3389/fnins.2022.998275 PMC9490374 36161165

[10] Duffner PK Barczykowski A Kay DM. Later onset phenotypes of Krabbe disease: results of the world-wide registry. Pediatr Neurol. 2012;46:298–306. 10.1016/j.pediatrneurol.2012.02.023 22520351

[11] Wenger DA Rafi MA Luzi P Datto J Costantino-Ceccarini E. Krabbe disease: genetic aspects and progress toward therapy. Mol Genet Metab. 2000;70:1–9. 10.1006/mgme.2000.2990 10833326

[12] Mikulka CR Sands MS. Treatment for Krabbe’s disease: finding the combination. J Neurosci Res. 2016;94:1126–37. 10.1002/jnr.23822 PMC5295787 27638598

[13] Richards S Aziz N Bale S. Standards and guidelines for the interpretation of sequence variants: a joint consensus recommendation of the American College of Medical Genetics and Genomics and the Association for Molecular Pathology. Genet Med. 2015;17:405–24. 10.1038/gim.2015.30 PMC4544753 25741868

[14] Chen S Jin J Xu ZQ Han H Wu L Li Z. Catalpol attenuates osteoporosis in ovariectomized rats through promoting osteoclast apoptosis via the Sirt6-ERα-FasL axis. Phytomedicine. 2024;123:155262. 10.1016/j.phymed.2023.155262 38100921

[15] Zhang W Cui N Su F. Serum, spleen metabolomics and gut microbiota reveals effect of catalpol on blood deficiency syndrome caused by cyclophosphamide and acetylphenylhydrazine. Front Immunol. 2023;14:1280049. 10.3389/fimmu.2023.1280049 PMC10655121 38022670

[16] Zhang MF Wang JH Sun S. Catalpol attenuates ischemic stroke by promoting neurogenesis and angiogenesis via the SDF–1α/CXCR4 pathway. Phytomedicine. 2024;128:155362. 10.1016/j.phymed.2024.155362 38522312

[17] Zhou S Chen X Gu X Ding F. Achyranthes bidentata Blume extract protects cultured hippocampal neurons against glutamate-induced neu-rotoxicity. J Ethnopharmacol. 2009;122:547–54. 10.1016/j.jep.2009.01.025 19429326

[18] Li X Wang J Gao L. Anti-inflammatory and analgesic activity of RAP (Radix Angelicae Pubescentis) ethanol extracts. Afr J Tradit Complement Altern Med. 2013;10:422–6. PMC3777581 24146469

[19] Yan Y Li S Li H Lin Y Yang J-X. Osthole protects bone marrow-derived neural stem cells from oxidative damage through PI3K/Akt-1 pathway. Neurochem Res. 2017;42:398–405. 10.1007/s11064-016-2082-y 27734182

[20] Zhang L Ravipati AS Koyyalamudi SR. Antioxidant and anti-inflammatory activities of selected medicinal plants containing phenolic and flavonoid compounds. J Agric Food Chem. 2011;59:12361–7. 10.1021/jf203146e 22023309

[21] Han X Ji X Zhao H. Mechanisms of coix seed compositions in the treatment of spleen deficiency and wet dampness zheng. Afr J Tradit Complement Altern Med. 2017;14:239–46. 10.21010/ajtcam.v14i4.26 PMC5471471 28638886

[22] Seo MJ Kim SJ Kang TH. The regulatory mechanism of β-eudesmol is through the suppression of caspase-1 activation in mast cell-mediated inflammatory response. Immunopharmacol Immunotoxicol. 2011;33:178–85. 10.3109/08923973.2010.491082 20604677

[23] Wang M Meng J Wang H Hu H Hong Y. Atractylodes macrocephala III suppresses EMT in cervical cancer by regulating IGF2BP3 through ETV5. J Cell Mol Med. 2024;28:e18081. 10.1111/jcmm.18081 PMC10868144 38358034

[24] Takase H Inoue O Saito Y Yumioka E Suzuki A. Roles of sulfhydryl compounds in the gastric mucosal protection of the herb drugs composing oren-gedoku-to(a traditional herbal medicine). Jpn J Pharmacol. 1991;56:433–9. 10.1254/jjp.56.433 1660547

[25] Liang Y Huang M Jiang X Liu Q Chang X Guo Y. The neuroprotective effects of berberine against amyloid β-protein-induced apoptosis in primary cultured hippocampal neurons via mitochondria-Related caspase pathway. Neurosci Lett. 2017;655:46–53. 10.1016/j.neulet.2017.06.048 28668383

[26] Wang Z-B Chen B-B Luo L Yan J-K. Fractionation, physicochemical char-acteristics and biological activities of polysaccharides from Pueraria lobata roots. J Taiwan Inst Chem Eng. 2016;67:54–60.

[27] Kim DH Lee JS Yun CY Kim D-H Kim IS. Chinese quince(Chaenomeles sinensis)extract inhibits cell migration and cytokine release in HMC-1 cells. Food Sci Biotechnol. 2013;22:501–6.

[28] He D-Y Dai S-M. Anti-inflammatory and immunomodulatory effects of Paeonia lactiflora Pall, a traditional Chinese herbal medicine. Front Pharmacol. 2011;2:10. 10.3389/fphar.2011.00010 PMC3108611 21687505

[29] Rigo FK Bochi GV Pereira AL. TsNTxP, a non-toxic protein from Tityus serrulatus scorpion venom, induces antinociceptive effects by suppressing glutamate release in mice. Eur J Pharmacol. 2019;855:65–74. 10.1016/j.ejphar.2019.05.002 31059709

[30] Zhang F Wu Y Zou XH Tang Q Zhao F Cao Z. BmK AEP, an anti-epileptic peptide distinctly affects the gating of brain subtypes of voltage-gated sodium channels. Int J Mol Sci. 2019;20:729. 10.3390/ijms20030729 PMC6387193 30744067

[31] Mikaelian AG Traboulay E Zhang XM. Pleiotropic anticancer properties of scorpion venom peptides:rhopalurus princeps venom as an anticancer agent. Drug Des Devel Ther. 2020;14:881–93. 10.2147/DDDT.S231008 PMC7051175 32161447

[32] Hsieh M-T Peng W-H Yeh F-T Tsai HY Chang YS. Studies on the anticonvulsive, sedative and hypothermic effects of periostracum cicadae extracts. J Ethnopharmacol. 1991;35:83–90. 10.1016/0378-8741(91)90136-2 1753798

[33] Wang JD Narui T Kurata H. Hematological studies on naturally occurring substances. II.Effects of animal crude drugs on blood coagulation and fibrinolysis systems. Chem Pharm Bull (Tokyo). 1989;37:2236. 10.1248/cpb.37.2236 2598329

[34] Song HY Han JM Byun EH Kim WS Seo HS Byun E-B. Bombyx Batryticatus protein-rich extract induces maturation of dendritic cells and Th1 polarization:a potential immunological adjuvant for cancer vaccine. Molecules. 2021;26:476. 10.3390/molecules26020476 PMC7831066 33477499

[35] Fan Y Nguyen TV Piao CH Shin HS Song CH Chai OH. Fructus Amomi extract attenuates nasal inflammation by restoring Th1/Th2 balance and down-regulation of NF-kappaB phosphorylation in OVA-induced allergic rhinitis. Biosci Rep. 2022;42:BSR20212681. 10.1042/BSR20212681 PMC8935377 35274678

[36] Zhang K Cao F Zhao Y Wang H Chen L. Antibacterial ingredients and modes of the methanol-phase extract from the fruit of Amomum villosum Lour. Plants (Basel). 2024;13:834. 10.3390/plants13060834 PMC10975419 38592864

[37] Mao R He Z. Pinellia ternata (Thunb) Breit: a review of its germplasm resources, genetic diversity and active components. J Ethnopharmacol. 2020;263:113252. 10.1016/j.jep.2020.113252 32798614

[38] Wang J Lyu CZ Zhao CR. Effects and mechanism of Setaria italica extract on improving sleep in insomnia mice. Chin J Pharm. 2024;35:322–6.

[39] Dang LL Jin YJ Yuan Y. Licorice: comprehensive review of its chemical composition, pharmacodynamics, and medicinal value. Acupunct Herb Med. 2024;4:136–50.

[40] Xu LW Xu S Wang J. Research progress on the pharmacological actions of herba siegesbeckia. J Changchun Univ Chin Med. 2021;37:704–8.

[41] Zhang Y Xiao FL Zhao XH. Research progress on chemical constituents and pharmacological activities of caulis spatholobi. Guangzhou Chem Ind. 2024;52:13–6.

[42] Stohr JR Xiao P-G Bauer R. Constituents of Chinese Piper species and their inhibitory activity on prostaglandin and leukotriene biosynthesis in vitro. J Ethnopharmacol. 2001;75:133–9. 10.1016/s0378-8741(00)00397-4 11297843

[43] Hijikata Y Kano T Xi L. Treatment for intractable anemia with the traditional Chinese medicines Hominis Placenta and Cervi Cornus Colla (deer antler glue). Int J Gen Med. 2009;2:83–90. 10.2147/ijgm.s5253 PMC2840555 20360892

[44] Ye J Yin P Xiao MT. New aromatic compounds from the rhizomes of Homalomena occulta. Phytochem Lett. 2017;21:57.

[45] Hu YM Liu C Cheng KW. Sesquiterpenoids from Homalomena occulta affect osteoblast proliferation, differentiation and mineralization in vitro. Phytochem. 2008;69:2367. 10.1016/j.phytochem.2008.05.023 18649899

[46] Zelik B Orhan I Aslan S. Antioxidant and antimicrobial actions of. the clubmoss Lycopodium clavatum L. Phytochem Rev. 2007;6:189–96.

[47] Chai SW Fan BS Song XJ. Research progress on pharmacological effects and clinical applications of mineral Chinese medicine in the treatment of insomnia. Tianjin J Tradit Chin Med. 2024;41:403–8.

[48] Xu B Yan Y Huang J Yin B Pan Y Ma L. Cortex Phellodendri extract’s anti-diarrhea effect in mice related to its modification of gut microbiota. Biomed Pharmacother. 2019;123:109720. 10.1016/j.biopha.2019.109720 31884345

[49] Li CG Yan L Jing YY. Berberine augments ATPinduced inflammasome activation in macrophages by enhancing AMPK signaling. Oncotarget. 2017;8:95–109. 10.18632/oncotarget.13921 PMC5352208 27980220

[50] Wang QY Bi YY Xu SJ. Progress of extraction methods and pharmacological effects of active components in angelica sinensis. Chem Reagents. 2025;47:43–51.

[51] Oh SH Kim SW Kim DJ. Sec-O-glucosylhamaudol mitigates inflammatory processes and autophagy via p38/JNK MAPK signaling in a rat neuropathic pain model. Korean J Pain. 2021;34:405–16. 10.3344/kjp.2021.34.4.405 PMC8494959 34593658

[52] Sun M Fan HT Jiang YY. Study on evaluation of immunoregulatory activity of polysaccharides from Fangfeng(Saposhnikovia divaricata)in vitro and in vivo. Chin Arch Tradit Chin Med. 2024;42:88–91+278–9.

[53] Kanauchi O Iwanaga T Andoh A. Dietary fiber fraction of germinated barley boodstuff attenuated mucosal damage and diarrhea, and accelerated the repair of the coloinc mucosa in an exper-imental colitis. J Gastroenterol Hepatol. 2001;16:160–8. 10.1046/j.1440-1746.2001.02427.x 11207896

[54] Liu YT Hu ZF. Effect of fiber extracted from germinated barley on intestinal microflora of rats with UC. Zhejiang J Integr Tradit Chin Western Med. 2008;18:471–2+8.

